# Visual occlusion effects on youth football players’ performance during small-sided games

**DOI:** 10.1371/journal.pone.0268715

**Published:** 2022-07-15

**Authors:** Sara Santos, Bruno Gonçalves, Diogo Coutinho, Gabriel Vilas Boas, Jaime Sampaio

**Affiliations:** 1 Department of Sports Sciences, Exercise and Health, University of Trás-os-Montes and Alto Douro (UTAD), Vila Real, Portugal; 2 Research Center in Sports Sciences, Health Sciences and Human Development, CIDESD, CreativeLab Research Community, Vila Real, Portugal; 3 University of Maia (UMAIA), Maia, Portugal; 4 Departamento de Desporto e Saúde, Escola de Saúde e Desenvolvimento Humano, Universidade de Évora, Évora, Portugal; 5 Comprehensive Health Research Centre (CHRC), Universidade de Évora, Évora, Portugal; 6 Portugal Football School, Portuguese Football Federation, Oeiras, Portugal; Universita degli Studi di Milano, ITALY

## Abstract

This study aimed to explore how youth players’ physical, technical and positional performance may be affected by visual occlusion when playing under different SSG pitch sizes. Under-15 players performed two experimental scenarios: a) normal situation, without visual occlusion; b) visual occlusion, by using an eye patch in the eye corresponding to the dominant foot. These scenarios were tested in a small (40x30m) and a larger pitch (50x35m). Players’ positional data was used to compute tactical and time-motion variables. In addition, technical analysis was comprised using video footage. Playing with visual occlusion in the larger pitch size induced higher distance covered while walking but lower running distance (*p <* .05). Although no statistically significant effects were identified between the normal and visual occlusion conditions for the tactical behaviour and technical performance a lower number of successful passes (small to moderate effect sizes) and higher regularity in the distance to the opponent’s team centroid (moderate effect size) were found with visual occlusion. Players covered more distance and achieved higher maximum speed in the larger compared to the small pitch (moderate to large effect size, *p <* .05), while also increasing their distance to both team’s centroid and increasing the regularity to these distances (moderate to large effect size, *p <* .05). Overall, despite similar effects for tactical and technical variables, some important practical information can be depicted. Accordingly, coaches may use the visual occlusion to promote more stable and regular behaviors while decreasing the physical demands. Larger pitches may be used to increase the distance between players’ and teams, as well as to induce higher physical load in both the normal and visual occlusion conditions. From the technical perspective, coaches may design smaller pitches to emphasize the use of the non-dominant foot during the occlusion scenario and promote the pass during the normal scenario.

## Introduction

Competing at a high level in team sports, such as association football, requires well developed perceptual skills [1]. Players’ decisions on the pitch are mediated by their ability to interact with the surrounding environment to unfold goal-directed behaviours [2, 3]. For that, players move their body, head and eyes to perceive the surrounding environment to properly guide their actions [4, 5]. Under this perspective, opportunities for action (i.e., affordances) will emerge as players move within the competitive environment [6]. This perspective highlights how important it is for players to visually explore the environment in the search for the best game options. Previous research has shown that the players who visually explore more the environment prior to receiving the ball are more likely to perform frontal passes [7] and with a higher percentage of success [8]. Considering the key role of the environment in guiding players’ actions, one primary aim for coaches is to design training tasks that assist them to explore the surrounding and identify the relevant game information [9–11].

Improving the players’ ability to scan the environment requires training tasks that may replicate the dynamic and unpredictable nature of competitive performance, such as the small-sided games (SSG), whereas they allow players to couple their actions to the environmental information [12]. Besides, SSG allows manipulating the rules (i.e., task constraints) to develop players specific behaviours [9, 10]. This view is grounded on the constraint-led approach (CLA) that emphasize the major role of manipulating task constraints to amplify the players’ attunement to environmental information [13]. Underpinned in this approach, there has been a growing interest in understanding how players adjust their movement behaviour due to different constraints manipulation in youth players [14, 15]. For instance, a study explored how players physical performance and technical actions were affected as a result of increasing the pitch size during a 4vs4 ball possession SSG in different age groups (under-11, U11; under-15, U15; and under-23, U23) [16]. In general, authors found higher physical demands in larger playing areas. Furthermore, the U11 performed more passes with both the dominant and non-dominant foot in larger playing areas, while the U23 increased their passing actions in smaller playing spaces [16]. These results may suggest that different opportunities for action emerge as the result of players perceptual and motor skills. This evidence has been consistently identified in different sports, such as volleyball, where female players from a higher level (i.e., regional) showed better scores in the technical and physical, as well in the cognitive function (measured with the Flanker task) compared to lower-level athletes (i.e., provincial) [17]. In addition, a recent study exploring the effects of playing with different types of balls (handball and rugby) compared to a football ball during a Gk+4vs4+Gk found higher physical demands and more shots performed with the handball ball, while shorter and more stable distances between teammates when playing with the rugby ball [18]. In this regard, the different physical features of the corresponding balls seem to vary the players’ opportunities for action. The aforementioned studies highlighted how changes in the environmental information as a result of constraints manipulation afforded the players towards distinct movement patterns.

Overall, it can be depicted that the football performance is highly dependent on the visual perceptual system to provide information for perceiving and acting [19]. The visual system provides a complex variety of information and understanding its role may add reliable information to support players’ game behaviour [20]. Under this scope, previous research exploring the role of visual occlusion (OCL), considering a more analytical approach, found distinct movement patterns as result of removing critical information during the action [21–23]. For example, one study intended to explore how youth football players dribbling time performance was affected by visual conditioning promoted by the Nike Vapor Strobe stroboscopic glasses (Nike Inc., Beaverton, Oregon, USA) [24]. In general, the results revealed an increase in the time needed to complete the task when performing under visual conditioning [24]. These findings suggest that when players’ visual feedback is temporarily occluded, they must slow down the movement due to removing critical information. Further, this study provided key information regarding how soccer players adjust their behavior as result of temporary OCL, however, different results may emerge when considering more dynamic and variable practice scenarios, such as the SSG situation. While considering a more realistic training scenario (i.e., passing task under different levels of difficulty), Dunton et al. [25] explored how players’ response accuracy and time in a passing task was affected by a training intervention where one group of players trained with googles and other performed the same intervention without it. The authors found improvements for the players using googles in all phases of the intervention. In this vein, it may be possible that designing training interventions embodied in OCL would enhance players’ ability to be fine-tuned with the environmental information. However, before this step, further research is required to understand how OCL may acutely impact players’ performance in game-based scenarios. This seems especially important if it is considered the lower levels of perceived motor competence by non-experienced players [26]. In fact, using perceptual-motor activities may contribute to improve the perceived motor competence, and consequently, contributing towards a better quality of life [26]. For instance, in lower levels of performance as those found in youth football, there seems to be a higher percentage of non-crossed laterality [27]. Under this perspective, by having the eye corresponding to the dominant foot occluded it may be possibly that players afford to use more often the non-dominant foot. However, further research is required to clarify this assumption. Therefore, this study aimed to explore the effects of playing with visual occlusion (OCL) during small-sided games performed under a smaller and a larger playing area in youth players’ physical, technical and positional performance. Considering that players need to explore the environment to sustain their actions, it was hypothesized that more regular positionings would emerge under the OCL condition, and consequently, lower physical demands compared to the NOR scenario. From the technical perspective, it was expected that players increased the number of touches on the ball while using more the non-dominant limb under the OCL condition, mainly during the small-pitch size. In addition, it is hypothesized that increasing the pitch size would amplify the differences between the NOR and OCL scenarios.

## Methods

### Participants

Ten youth football players (age = 13.9 ± 1.6 years; Height = 163.1 ± 6.1 cm; body mass = 56.2 ± 4.2 kg; Years of experience = 6.1 ± 0.9 years) from a U15 soccer academy participated in this study. All players performed 4 training sessions (90–115 min) per week and played an official eleven-a-side game during the weekend at standard regular football field (104 × 64 m). The goalkeepers’ positioning is very restricted to a specific area, and their positioning dynamics are different from the outfield players. For this reason, the goalkeepers were excluded from the analysis. A written and informed consent was provided to the coaches, players, and by their legal guardians, as well as by the club, before the beginning of the study. All participants were notified that they could withdraw from the study at any time. The study protocol followed the guidelines and was approved by the local Ethics Committee of the Research Center in Sports Sciences, Health Sciences and Human Development (UIDB/4045/2020) and conformed to the recommendations of the Declaration of Helsinki.

### Study design

**Fig 1:**
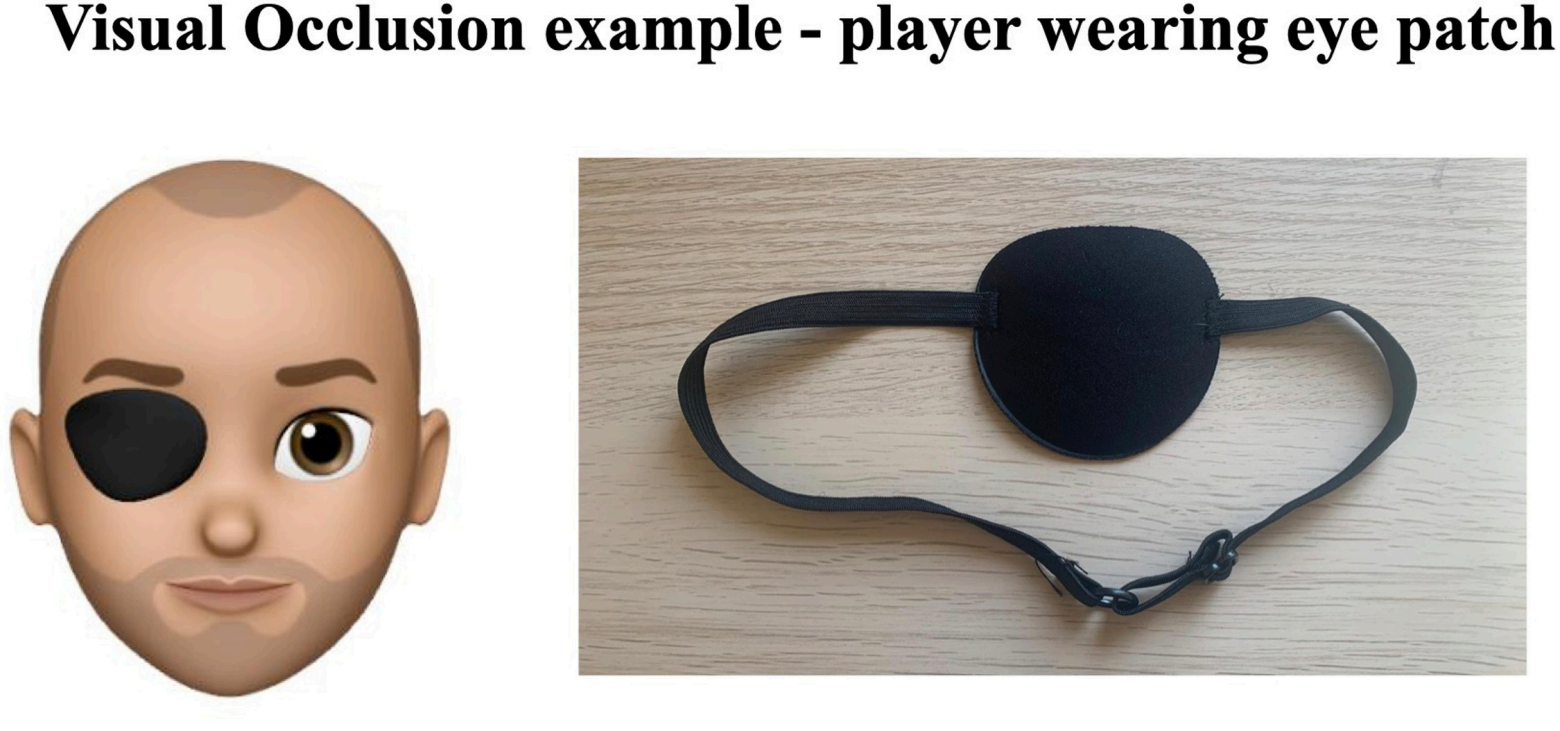
Representation of a player wearing the eye patch during the SSG.

**Fig 2:**
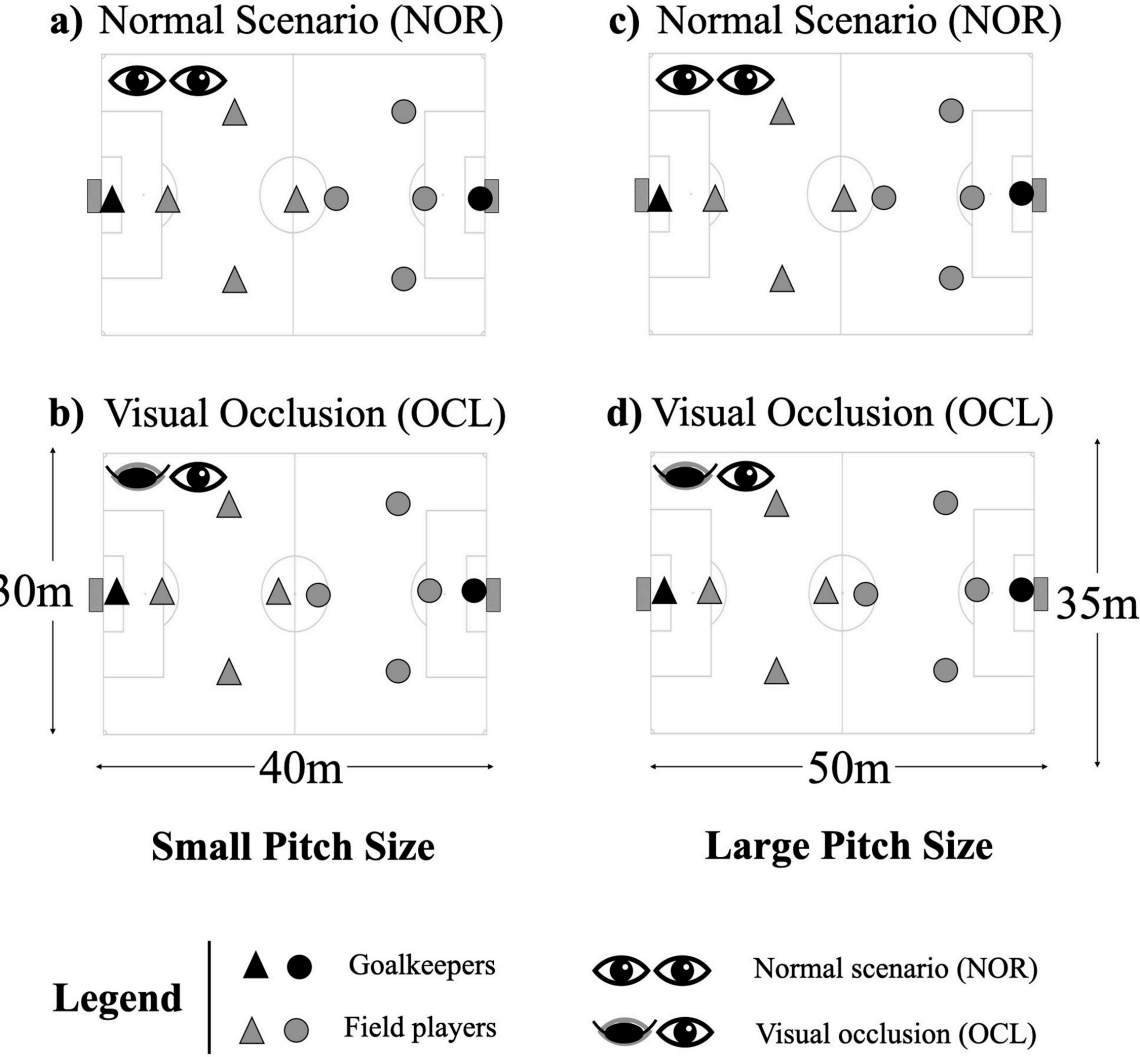
(a) representation of the experimental scenarios (normal—N; visual occlusion–VO); (b) schematic representation of data collection design.

The study design was based on a repeated-measures approach, where players were exposed to two different experimental scenarios under two different pitch sizes. Accordingly, players performed a Gk+4vs4+Gk SSG with the following experimental scenarios: (i) normal condition (NOR) performed in a small-pitch size (40x30m, Fig 2A), where the SSG was performed without visual occlusion; (ii) visual occlusion (OCL), in which the players from both teams wear an eye patch occluding the eye corresponding to the dominant foot (see Fig 1) during a SSG performed in a small-pitch size (40x30m, Fig 2B). In addition, previous research has shown that youth players’ opportunities for action are modified as resulting of changes in the pitch size [16]. Therefore, players were also exposed to: (iii) normal condition in a larger pitch size (50x35m, Fig 2C); and (iv) visual occlusion in a large pitch size (50x35m, Fig 2D). Altogether, it was possible to inspect the following conditions: a) effects of increasing the pitch size without visual occlusion; b) effects of increasing the pitch size with visual occlusion; c) effects of playing with visual occlusion in a small pitch size; d) effects of playing with visual occlusion in a large pitch size.

### Procedures

The testing procedures were performed across four sessions on non-consecutive days. The first session was used to familiarize the players with the experimental scenarios by exposing them to two bouts of 5-min. Further, three testing sessions were used to assess players’ performance. On the first testing day, the head coach selected the eight best players from the team according to their technical, tactical, and physical skills and distributed them into two balanced teams considering their playing positions. Before the SSG, there was a standardized 25-min warm-up composed of low intensity running, limb activation, dynamic stretching exercises and ball possession exercises (4-a-side without goals). After the warm-up, the players were first exposed to the small pitch and then to the large pitch. In each pitch, the players performed two bouts of 5-min interspersed with 2-min of rest whereas a 10-min rest was provided between pitch scenarios. Therefore, each player participated in three bouts of 5-min of each experimental scenario on each pitch size, accounting for a total of 12 SSG bouts. The SSG were performed on an artificial turf pitch. The players were encouraged to hydrate with water before the SSG and in-between the bouts. Apart from the offside rule that was not applied and the restart of the game by the goalkeeper that conceded a goal to ensure a fast restart, all the remaining rules were played according to the FIFA football rules.

### Data collection

Positional data and the distance covered during SSG were gathered using 5 Hz global positioning system (GPS) units (SPI-PRO, GPSports, Canberra, ACT, Australia). The units were placed into appropriate elastic harnesses that placed the device on the upper back of each participant. The players’ latitude and longitude coordinates obtained with the GPS units were resampled to remove possible data gaps and synchronize all the individual data. Following this procedure, the data were converted to meters using the Universal Transverse Mercator (UTM) coordinate system and a rotational matrix to adjust the players’ displacement data, pitch length and width with the appropriate x and y-axis. This procedure was carried out by the data retrieved from four GPS units placed on each pitch corner [28]. The positional data of the players were used to determine the following variables: (i) distance from each player to the team centroid (the team centroid as measured by the mean position from all outfield players) and distance from each player to the opponent team centroid (the opponent team centroid as measured by the mean position from all opponent outfield players). These variables may provide functional information about how players’ decision-making (positioning related) is more based on perceived information from their teammates/opponents. Also, it may provide functional information about team structures since reflect the contraction/dispersion of the teams. The variables were computed with linear and nonlinear processing technique. The linear variables were absolute values (reported in meters) and the corresponding coefficient of variation (CV, i.e., the magnitude/amplitude of the variability). The nonlinear variable was the approximate entropy (ApEn). ApEn computation technique has been used to quantify the structure of the variability from a specific time-series. Input values for computations were 2.0 to the vector length (m) and 0.2 standard deviations to the tolerance factor (r) [29]. The outcome range between 0 and 2 (arbitrary units) and lower values represented more repeatable, regular, predictable and less chaotic sequences of data points [30, 31]. From a processing approach, ApEn expresses the probability that the configuration of one segment of data in a time series will allow the prediction of the configuration of another segment of the time series a certain distance apart. In practice, this technique may be used, for example, to identify if players’ positioning dynamics express a regular and predictable pattern which may, in turn, provide information regarding their tactical behaviour [32]. The total distance covered, distance covered at different movement speed categories were used as external workload variables. The following speed categories were used: walking (0–6.9 km/h); light jogging (7.0–9.9 km/h); faster jogging (10.0–12.9 km/h); running (13.0–15.9 km/h); sprinting (16.0–17.9 km/h); and maximal speed (>18.0 km/h) [33].

The SGG were recorded using a digital video camera, Panasonic NV-GS230, that was fixed at a 2-m height and aligned in the midfield part of the pitch. Then, the video files were downloaded to a computer and a notational analysis was performed using the LongoMatch software (Longomatch, version 1.3.7., Fluendo) [18]. The following individual performance variables were collected: i) the total number of shots on and off-target while considering the dominant and non-dominant foot; ii) number of successful and unsuccessful dribbles; iii) number of ball touches when considering the dominant and non-dominant foot; iv) the total number of successful and unsuccessful passes while considering the dominant and non-dominant foot.

### Statistical analysis

Descriptive results are presented as mean ± standard deviation. A repeated-measures analysis of variance was performed to identify inferential differences in considered variables according to the conditions: without visual occlusion–normal (NOR) and visual occlusion (OCL); and pitch size–small and big pitch. When appropriate the Tukey HSD was used for *post-hoc* comparisons. Statistical significance was set at *p* < 0.05 and calculations were completed using the Jamovi Project (Computer Software Version 1.2, 2020). The scenarios NOR performed in small pitch vs OCL performed in big pitch; and NOR performed in small pitch vs OCL performed in small pitch were not included in results and discussion, once they are not relevant for the study purpose. The Cohens’ *d* was computed as effect size (ES) and the interpretation for the standardized effect size was based on the following criteria: 0.2, trivial; 0.6, small; 1.2, moderate; 2.0, large; and >2.0, very large [34].

## Results

### Effects of playing with OCL in smaller pitches

**Fig 3:**
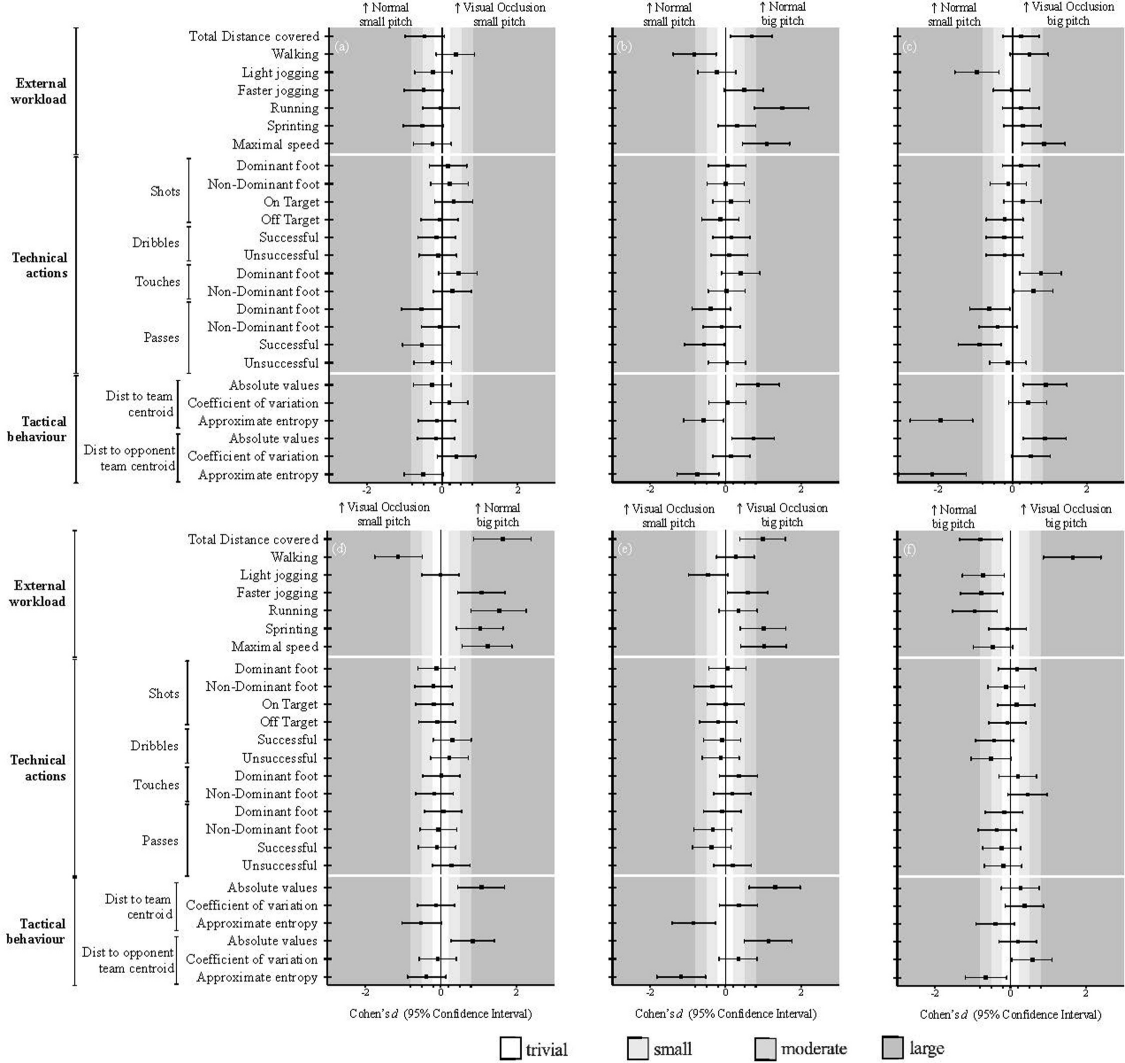
Standardized (Cohen) differences in the tactical behaviour, external workload and technical actions variables according to game scenarios (N vs VO) and pitch size (small vs big). Error bars indicate uncertainty in the true mean changes with 95% confidence intervals.

The differences between NOR and OCL in the small pitch can be depicted in Table 1 and Fig 3A. Although the results did not show any statistical significance difference, some trends can be identified from the ES (Cohen *d*). Accordingly, in general, lower external workload values were identified when playing with OCL, whereas it was found lower values of total distance covered (small ES), faster jogging (small ES) and sprinting (moderate ES). In addition, while it was found a higher number of touches with the dominant foot (small ES) during the OCL condition, lower values of successful passes (moderate ES) were found in this condition compared to the NOR scenario. From the tactical behaviour, lower values of ApEn (i.e., higher regularity) in the distance to the opponent team centroid (moderate effects) were found with OCL.

**Table 1:** Descriptive and inferential analysis of players’ performance measures according to the condition (NOR vs OCL) and pitch size (small vs big).

Variables	Game Scenarios (mean±SD)				*F*	*p-value*	*Post hoc*
	Normal (NOR) small pitch	Visual Occlusion (OCL) small pitch	Normal (NOR) big pitch	Visual Occlusion (OCL) big pitch			
Total Distance covered (m)	501±63.4	475±47.2	551±63.8	514±59.3	11.1	< .001*	b|d|e
Walking (<7.0 Km/h)	227±19.2	232±16.4	211±22.2	240±27.7	10.1	< .001*	b|d|f
Light jogging (7.1–10.0 km/h)	111±28.8	104±34.9	103±24.9	86.9±18	3.55	.022*	c
Faster jogging (10.1–13.0 km/h)	84.6±33.8	71.5±23.6	103±31	84.1±28.5	6.46	< .001*	d
Running (13.1–15.0 km/h)	43.8±16.4	43.2±17.8	71.8±20.4	50.5±30.3	12.4	< .001*	b|d|f
Sprinting (15.1–8.0 km/h)	19.3±10.4	11.5±9.2	24.3±13	23.4±12.5	5.76	.002*	d|e
Maximal speed (>18.1 km/h)	15.3±13.9	12.7±11.8	38.1±20.7	29.5±16.5	15	< .001*	b|c|d|e
**Shots**							
Dominant foot	0.9±0.7	1.1±1.16	0.9±0.9	1.1±1,0	0.304	.823	-
Non-Dominant foot	0.3±0.4	0.3±0.5	0.2±0.4	0.1±0.3	0.56	.644	-
On Target	0.6±0.7	0.9±0.9	0.7±0.9	0.9±0.9	0.699	.558	-
Off Target	0.5±0.6	0.4±0.5	0.4±0.5	0.3±0.6	0.33	.803	-
**Dribbles**							
Successful	1±1.21	0.81±1.11	1.3±1,0	0.7±1.1	0.966	.417	-
Unsuccessful	0.69±0.95	0.56±0.81	0.8±0.8	0.4±0.6	0.743	.532	-
Touches							
Dominant foot	10.1±5.3	11.9±6.3	12.0±8.1	13.4±7.5	2.35	.085	-
Non-Dominant foot	2.2±1.6	2.8±1.8	2.3±1.5	3.3±2.2	1.35	.27	-
**Passes**							
Dominant foot	5.9±2.9	4.5±2.3	4.7±2.2	4.3±2.3	2.37	.083	-
Non-Dominant foot	0.8±0.9	0.7±0.9	0.6±0.7	0.3±0.5	1.06	.376	-
Successful	5.8±2.9	4.7±2.3	4.4±2.2	3.9±1.9	3.89	.015*	c
Unsuccessful	0.9±1.3	0.5±0.7	0.9±1.1	0.7±0.8	0.597	.62	-
**Distance to own team centroid**							
Absolute values (m)	6.1±1.2	5.9±0.9	7.1±1	7.4±1.3	12.8	< .001*	b|c|d|e
Coefficient of variation (CV)	0.45±0.07	0.46±0.07	0.45±0.07	0.48±0.05	1.23	.311	-
Approximate entropy (ApEn)	0.23±0.05	0.22±0.04	0.2±0.04	0.17±0.04	7.48	< .001*	c|e
**Distance to opponent team centroid**							
Absolute values (m)	6.8±1.4	6.7±1.3	8.0±1.4	8.2±1.3	10.8	< .001*	b|c|d|e
Coefficient of variation (CV)	0.45±0.11	0.47±0.09	0.46±0.06	0.5±0.07	2.09	.114	-
Approximate entropy (ApEn)	0.27±0.05	0.24±0.04	0.22±0.05	0.19±0.03	13.9	< .001*	b|c|e

Abbreviations: OCL = visual occlusion; CV = coefficient of variation; ApEn = approximate entropy.

*p < .05

Post hoc differences: a) NOR in the small pitch vs OCL in the small pitch; b) NOR in the small pitch vs NOR big pitch; c) NOR in the small pitch vs OCL in the big pitch; d) OCL in the small pitch vs. NOR in the big pitch; e) OCL in the small pitch vs OCL in the big pitch; f) NOR in the big pitch vs OCL in the big pitch

### Effects of increasing the pitch size during the normal scenario

Table 1 and Fig 3B presented the effects of increasing the pitch size when playing in a N scenario. From the external workload perspective, higher demands were found in general when performing in the big pitch size, whereas it was found differences for the total distance covered (*p <* .05, moderate ES), walking distance (*p <* .05, large ES), running distance (*p <* .05, large ES) and maximum speed (*p <* .05, large ES). From the technical perspective, the results did not show statistically significant differences between the conditions. However, a higher number of touches with the dominant foot (small ES) in the bigger pitch size was identified, while in turn, the players performed a lower number of passes with the dominant foot (small ES) and number of successful passes (moderate ES). From the tactical behaviour, higher values in the distance to both the team (*p <* .05, large ES) and opponent team centroid (*p <* .05, moderate ES) were identified during the big pitch size, while lower ApEn values in the distance to the opponent team centroid (*p <* .05, moderate ES).

### Effects of increasing the pitch size during the OCL scenario

The effects of increasing the pitch size when playing with OCL in the external workload, technical performance and tactical behaviours can be found in Table 1 and Fig 3E. When taking into consideration the external workload, statistically significant differences were identified for the total distance covered (*p <* .05, large ES), sprinting distance (*p <* .05, large ES) and maximum speed (*p <* .05, large ES), with higher in the big pitch. No statistically differences were observed for the technical performance. However, some results might be identified from the ES, where it can be identified higher values for the non-dominant number of shots (small ES) and passes (small ES), as well as in the number successful passes (small ES) for the small pitch. From the tactical variables, statistically differences were found with higher values in big pitch regarding the distance to both the team centroid (*p <* .05, large ES) and opponent team centroid (*p <* .05, large ES), and lower values in the regularity (i.e., ApEn) values in the distance to the team (*p <* .05, large ES) and opponent team centroid (*p <* .05, large ES).

### Effects of playing with OCL in larger pitches

The differences between playing NOR vs OCL in the big pitch can be depicted from Table 1 and Fig 3F. While higher values in the walking distance (*p <* .05, large ES) were found during the OCLcondition, in turn, players covered higher values while running (*p <* .05, large ES) during the NOR condition. The results from the ES also allowed to depict lower values of successful (small ES) and unsuccessful dribbles (small ES) during the OCLscenario compared to the NOR condition. In addition, lower ApEn values in the distance to the team (small ES) and opponent team centroid (moderate ES) were found when playing with OCL.

## Discussion

This study aimed to explore how youth players’ physical, technical and positional performance may be affected by visual occlusion when playing under different SSG pitch sizes. Not confirming our hypothesis, results showed no significant effects were identified between the tactical and technical performance when comparing the N with the OCL condition, indicating that players can adjust their behaviour when playing under such constraint. Despite that, and following our hypothesis, more stable and regular positioning were identified when playing with OCL, resulting in a decrease in the physical demands. In addition, as it was expected, higher differences between conditions were found in the large pitch size, while the small pitch size amplify the use of the non-dominant foot.

### Effects of playing with OCL in small pitches

The results showed a higher regularity in the distance to the opponents’ centroid when playing with OCL. This result suggests that the players may rely more on their opponents’ positioning when performing with OCL. Accordingly, it may allow them to perceive the opponents positioning when in possession, providing information regarding how much space and time would have to decide. On the other hand, playing with OCL without possession, the players may be more focused on the local information (i.e., direct opponent) and less on the teammates, and maintaining more regular distances possibly allow them to pressure while defending. This strategy allows to anticipate the opponents’ movement and explore adaptive movement patterns [35–37]. There was a trend towards lower physical demands and an increase in the number of touches with the dominant foot when playing with OCL. These results may be linked with the lower ability to scan the environment when playing with OCL and the shorter distances between opposing teams when performing in smaller pitches. That is, when under possession with OCL, the player may not be able to properly scan the environment while suffering pressure from the opponent, and so the player may have to continuously touch the ball with the dominant foot to protecting it in order to find opportunities for action. As result, it is likely that it decreases the game pace as the players might stay more time on possession while trying to find possible passing actions.

### Effects of increasing the pitch size during the N scenario

The effects of increasing the pitch size have been widely explored by the available literature [16, 38–40]. Accordingly, increasing the pitch size have contributed to higher distance between players from the same team and to opposing players [40]. Similar findings were identified in this study, since higher distance to both team’s centroid and opponents’ team centroid were found in the larger pitch size. In addition, we also found higher total and distance covered at high intensities during the larger pitch, possibly as result of the increase in the available space to perform [38, 39]. From the technical perspective, in general there were more average touches with the dominant foot in the larger pitch size, whereas emerged less successful passes and passes performed with the dominant foot. Smaller pitch sizes seems to increase the spatial-temporal pressure on the player under ball possession due the shorter distances to the opponents [39, 40] and players may afford to pass more often to limit the defensive players’ chances to press and recover the ball. In contrast, the higher distance to the opponents as well as the higher available space during larger pitch sizes, may afford the players to touch more often on the ball to keep its possession while exploring the environmental information towards to support their actions.

### Effects of increasing the pitch size during the OCL scenario

Players seems to increase their distance to both teammates and opponents’ when playing in larger spaces [40]. Similarly, this study found an increases in these distances when playing under OCL. Larger pitch sizes have been used to increase the task physical demands [38, 39], and similar findings were identified in this study as higher total distance covered and distance covered while sprinting were found in this scenario. Further, higher maximal speed were identified during the larger pitch size, which may result not only from the higher available space, but also the higher distances between players from both teams affords players to cover a higher distance without requiring changes-of-direction, contributing to higher speed values [39]. Nevertheless, it is important to note that these values were below from those identified when playing without OCL. Despite not present statistically significant differences, some trends were identified in the technical actions. In general, presented average of shots and passes performed with the non-dominant foot, as well as a higher number of successful passes in the smaller pitch. Past research has shown increases in the number of shots as result of the closer distance to target [38, 39], and in the number of passes due to the lower distances to the opponents which increases the pressure on the ball [39]. However, in this study these increases in the average values were related with actions performed with the non-dominant foot. The eye patch was used in the eye corresponded to the dominant foot, which may reduce the players ability to capture information from the related side. Despite the intention of using the preferred foot, the smaller pitch size decreases the available space and time to decide as result of the lower distance to the opponents, and so, using the non-dominant foot may emerge as a functional movement behaviour. In contrast, during large pitch sizes, the distance to the opponents increases, which may allow more time for the players to use more often the dominant foot.

### Effects of playing with OCL in bigger pitches

Increasing the pitch size when in interaction with other constraints seems to amplify the effects when compared to small pitch sizes [16]. In this study, we also found a higher magnitude in the differences when comparing the OCL with the NOR scenario during the larger pitch size. Accordingly, the differences in the distance covered become more evident in the larger pitch size, emphasizing that when performing with OCL, the players’ decrease the game pace and movements on the pitch, possibly, to have more time to fine-tuned within the environmental information. In fact, it seems that the players ability to perform in fast-paced sports, such as football, is affected by the amount of available information that players’ can use through the visual feedback [24]. Further, the results showed more regular distances according to both team and opponents’ centroid, which may suggest that adopt more stable and regular positioning when playing with OCLand as result, their physical demands decrease. Despite the lack of statistical significance, it was identified lower number of dribbles performed, both successful and unsuccessful during the OCL scenario. These results are in line with those found during an analytical motor task based on the dribbling action, which found poor performances with OCL [24]. Accordingly, to successfully dribble, the players must retrieve information from the available space, the ball and the direct opponents’ body orientation [37], information that may be more difficult to capture when playing with OCL.

Despite the important and practical findings that resulted from this study, some limitations may be acknowledge. The lower sample size may refrain from achieving stronger inferences. Taking this into consideration, future studies may should consider a larger sample, but also incorporate players with distinct previous experiences and players belonging to different levels of performance (i.e., amateurs, semi-professionals, professionals, differences between males and females), since it’s a variable that interact differently with the information. Also, the eye patch was used in the eye corresponding to the dominant foot, and different results may have emerged if it was explored the interaction of the eye patch location (e.g., using it in the opposing side of the preferred foot). In addition, this study was based on acute effects, and considering that players seem to beneficiate from more long-term interventions sustained on OCL, it would also be interesting to explore the long-term effects of training with OCL in players performance during SSG. Therefore, future studies may consider training intervention grounded on the use of OCL, while considering different samples expertise.

## Conclusions

In general, no statistically significant differences were found between conditions (N and VO) for the technical and positional performance, not confirming our initial hypotheses. Nevertheless, some important and practical differences between conditions were depicted. Accordingly, playing with OCL may limit the players ability to interact with surround information, as it was found a more stable positioning, lower physical performance, and a higher number of touches on the ball under this constraint. Further, while using the dominant foot emerges more often during the NOR condition, the OCL seems to stress the use of the non-dominant foot, mainly in the smaller pitch size, possibly as result of the lower distance to the opponents. In contrast, larger pitch sizes seems to emphasize the distance between players while also increase the physical demands in both comparisons (NOR small vs NOR large; and OCL small vs OCL large). Based on these results, coaches may increase the pitch size to promote team separateness while increasing the physical demands. Adopting larger pitches with OCL may allow the players to better adjust their behaviours according to the spatial-temporal demands. In contrast, smaller pitch sizes may be used to emphasize the passing action under the NOR scenario, while develop players’ ability to use the non-dominant foot during the OCL scenario.
